# Novel Gold Nanoparticles Reduced by *Sargassum glaucescens*: Preparation, Characterization and Anticancer Activity

**DOI:** 10.3390/molecules21030123

**Published:** 2016-03-01

**Authors:** Zahra Ajdari, Heshu Rahman, Kamyar Shameli, Rasedee Abdullah, Maaruf Abd Ghani, Swee Yeap, Sahar Abbasiliasi, Daniel Ajdari, Arbakariya Ariff

**Affiliations:** 1 Innovation Center for Confectionery Technology (MANIS), Faculty of Science and Technology, Universiti Kebangsaan Malaysia, 43600 Bangi, Selangor Darul Ehsan, Malaysia; z_azhdari@yahoo.com (Z.A.); maaruf71@ukm.edu.my (M.A.G.); 2 Darou Pakhsh Pharmaceutical Manufacturing Company, St 61th Daroupakhsh City, Km 18- Karaj Hiway, 1411816616 Tehran, Iran; 3 Faculty of Veterinary Medicine, Universiti Putra Malaysia, 43400 UPM Serdang, Selangor, Malaysia; heshusr77@gmail.com (H.R.); rasedee@gmail.com (R.A.); 4 College of Veterinary Medicine, University of Sulaimani, Street 11, Zone 207, Sulaimani Nwe, 00964 Sulaimani City, Kurdistan Region, Iraq; 5 Malaysia-Japan International Institute of Technology, Universiti Technology Malaysia, Jalan Sultan Yahya Ahmad Petra, 54100 Kuala Lumpur, Malaysia; kamyarshameli@gmail.com; 6 Institute of Bioscience, Universiti Putra Malaysia, 43400 UPM Serdang, Selangor, Malaysia; skyeap2005@gmail.com; 7 Department of Bioprocess Technology, Faculty of Biotechnology and Biomolecular Science, Universiti Putra Malaysia, 43400 UPM Serdang, Selangor, Malaysia; upmsahar@yahoo.com; 8 Iranian Fisheries Research Organization, No. 297, West Fatemi Avenue, P. O. Box 14155-6116, 1411816616 Tehran, Iran; danielajdari@yahoo.com

**Keywords:** *Sargassum glaucescens*, gold nanoparticles, anticancer properties, cervical cancer, *in vitro* study

## Abstract

The current study investigated the anticancer properties of gold nanoparticles (SG-stabilized AuNPs) synthesized using water extracts of the brown seaweed *Sargassum glaucescens* (SG). SG-stabilized AuNPs were characterized by ultraviolet-visible spectroscopy, transmission and scanning electron microscopy, and energy dispersive X-ray fluorescence spectrometry. The SG-stabilized AuNPs were stable and small at 3.65 ± 1.69 nm in size. The *in vitro* anticancer effect of SG-stabilized AuNPs was determined on cervical (HeLa), liver (HepG2), breast (MDA-MB-231) and leukemia (CEM-ss) cell lines using fluorescence microscopy, flow cytometry, caspase activity determination, and MTT assays. After 72 h treatment, SG-stabilized AuNPs was shown to be significant (*p* < 0.05) cytotoxic to the cancer cells in a dose- and time-dependent manner. The IC_50_ values of SG-stabilized AuNPs on the HeLa, HepG2, CEM-ss, MDA-MB-231 cell lines were 4.75 ± 1.23, 7.14 ± 1.45, 10.32 ± 1.5, and 11.82 ± 0.9 μg/mL, respectively. On the other hand, SG-stabilized AuNPs showed no cytotoxic effect towards the normal human mammary epithelial cells (MCF-10A). SG-stabilized AuNPs significantly *(p <* 0.05*)* arrest HeLa cell cycle at G2/M phase and significantly *(p <* 0.05*)* activated caspases-3 and -9 activities. The anticancer effect of SG-stabilized AuNPs is via the intrinsic apoptotic pathway. The study showed that SG-stabilized AuNPs is a good candidate to be developed into a chemotherapeutic compound for the treatment of cancers especially cervical cancer.

## 1. Introduction

A report published in 2011 by the World Health Organization (WHO) placed cancer as the third leading cause of death, after heart disease and stroke, in most developed countries and the second leading cause of death, after heart disease, in Malaysia [1]. Due to the gravity of the disease, since 2007, the National Cancer Institute in Malaysia has approved approximately RM5 billion in funds for cancer research. This amount does not include funds provided by other federal agencies, states, local governments, voluntary organizations, private institutions, and industries. Despite the significant investments in cancer research, the overall mortality has nevertheless remained the same, suggesting the contribution of research to the alleviation of the disease has been minimal. Thus, there is pressing need for the discovery of more sensitive, accurate, and cost-effective methods for detecting and treating cancer [2].

Surgery, chemotherapy, and radiation therapy are the conventional strategies for the treatment of cancers. Most of these treatments are plagued with side-effects that further deteriorate the quality of life of patients under treatment. Current treatment regimens are non-specific and adversely implicate healthy tissues and organs with their effects. In the search for more specific and less harmful treatment methods, nanomedicine and nanoparticle drug-delivery were shown to be promising as alternatives for current treatment regimens. The role nanotechnology can play in medicine is enormous, potentially in the early detection, accurate diagnosis, and personalized treatment of cancers [3]. Nanoparticle drug-delivery can in fact be manipulated to provide properties for targeting diseased tissues and specificity. What also makes nanostructures particularly attractive are their versatility in their features to include nanoscaling, unique optical, electronic, and magnetic properties and their production, which can easily be up-scaled. These unique characters of nanostructures have opened new realms in the field of medicine [4,5,6].

Nanoparticles are smaller in size than some biological molecules, including lipids, nucleic acids, and proteins. The size of nanoparticles lies between one hundredth to one ten-thousandth of the size of human cells and they have incredible and extraordinary interactions with biomolecules within and on the surfaces of cells [7]. Since these properties can potentially be beneficial in medical applications, over the last decade, research in nanotechnology had grown exponentially and many nanotechnology centers have begun to appear all over the world [8,9].

Numerous nanoparticles have been synthesized, including those of noble metals such as gold (Au). Since molecular imaging mainly depends on the detection of specific biomarkers, the presence of Au nanoparticles bound to specific ligands on tissue cells can be a means of detection of diseased tissues like tumors [2]. Even with this knowledge, however, metal nanoparticles still find more industrial than medical applications [10].

Gold is a rare metallic element with a melting point of 1064 °C and a boiling point of 2970 °C. In 1890, the application of gold in modern medicine began to be realized when the German bacteriologist Robert Koch discovered that gold cyanide is bacteriostatic to the tubercle bacillus [11]. This subsequently led to the use of gold in the treatment of tuberculosis in the early 20th century [12].

The synthesis of nanoparticles can be achieved by methods such as physical, chemical, biological, and hybrid techniques that result in different types of nanoparticles [13,14]. Recently, it was discovered that gold and silver nanoparticles can be synthesized from various plant sources, using methods now broadly called green synthesis, that results in relatively small nanoparticles of sizes ranging from 20 to 30 nm in diameter [12,13,15]. Green synthesis of nanoparticles is a welcome advancement over chemical and physical methods as it opens the doors for cost-effective, environmentally friendly and easy to scale-up procedures without the need for high pressure, energy, temperature or toxic chemicals [16].

Some aquatic resources are now being used for the production of metal nanoparticles and this technique is safe, eco-friendly, and provides pure products. Among the aquatic resources used for nanoparticle production are seaweeds, commercially important marine living renewable resources [17], widely accepted as healthy functional foods due to their rich content of lipids, minerals, vitamins, polysaccharides, proteins, and polyphenols. Seaweed has been shown to have potential in the treatment of oxidative stress diseases, inflammation, allergies, diabetes, thrombosis, obesity, lipidemia, and hypertensive and other degenerative diseases. Naturally occurring seaweed also contains anti-tumour compounds [18,19,20].

**Figure 1:**
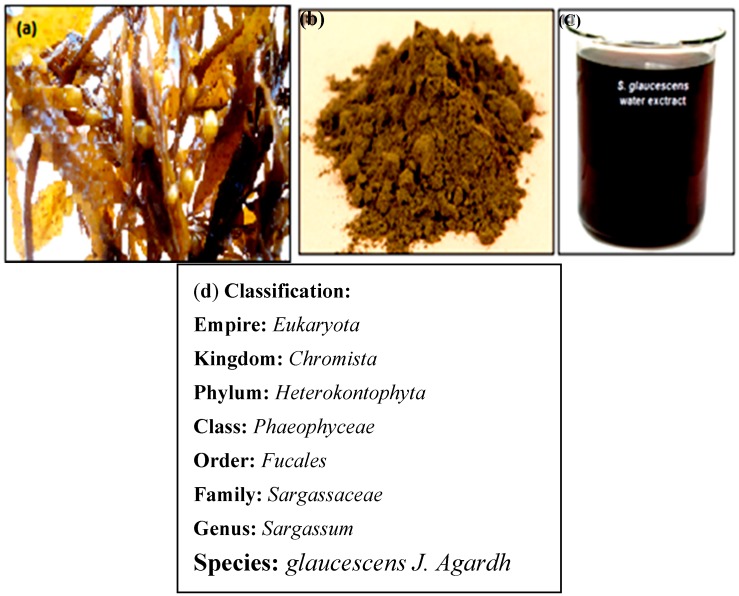
(**a**) Fresh SG seaweed; (**b**) SG dry powder; (**c**) SG water extract; (**d**) SG classification.

Currently, the cytotoxic effects of gold nanoparticles (AuNPs) are not clear and the effect of the nanoparticles on cell viability and propagation are essentially unknown [21]. Thus, the current study describes a green and rapid method of synthesizing a novel gold nanoparticles from brown seaweed *Sargassum glaucescens* (SG) (Figure 1) water extract (SG-stabilized AuNPs). The *in vitro* anti-cancer activity of the resulting SG-stabilized AuNPs was also examined using several cancer cell lines.

## 2. Results

### 2.1. Preparation of SG-Stabilized AuNPs

**Figure 2:**
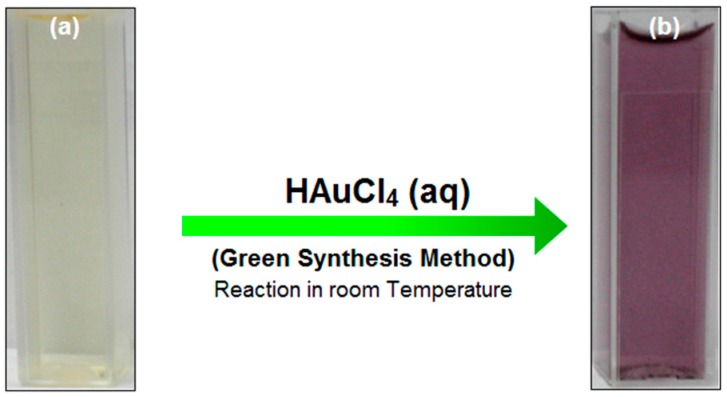
(**a**) SG water extract; (**b**) Au/SG suspension after 4 h of stirring.

Reduction of Au ions into AuNPs after exposure to the water extract of *Sargassum glaucescens* (SG) seaweed powder can be followed by the color change. The fresh SG seaweed emulsion is yellowish brown in color and after adding HAuCl_4_ and stirring for 4 h at room temperature, the emulsion turns into a reddish suspension within a few min (Figure 2a,b). The production of the reddish suspension after treatment of SG seaweed suspension with HAuCl_4_ also serves as an identification method for seaweed plants that can potentially be used for AuNPs synthesis.

### 2.2. SG-Stabilized AuNPs Characterization

#### 2.2.1. Ultraviolet-Visible (UV-Vis) Spectroscopy Analysis

**Figure 3:**
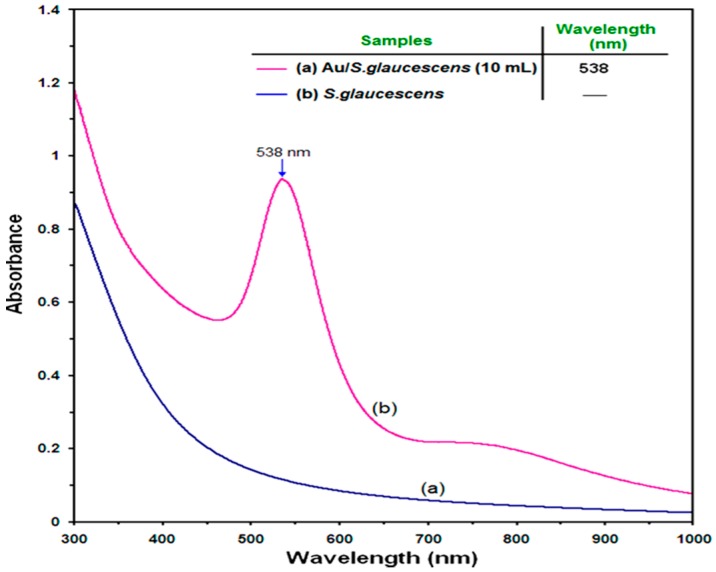
UV–Vis absorption spectra of SG (**a**); Au/SG suspension (**b**) after 4 h of stirring time.

The SG and Au/SG suspension was subjected to surface plasmon resonance (SPR) over the wavelength range from 300–1000 nm (Figure 3a,b). The SPR bands are influenced by the size, shape, morphology, composition and the dielectric environment of the prepared nanoparticles. Previous studies have shown that the spherical AuNPs contribute to the absorption bands at around 540–520 nm in the UV-visible spectra [22]. These absorption bands result from the AuNPs extra-fine nature and small size. The UV-Vis absorption spectra (Figure 3b) showed that the broad SPR band contained one peak at 538 nm that illustrates the presence of a homogeneous distribution of hydrosol AuNPs.

#### 2.2.2. Morphology Study

**Figure 4:**
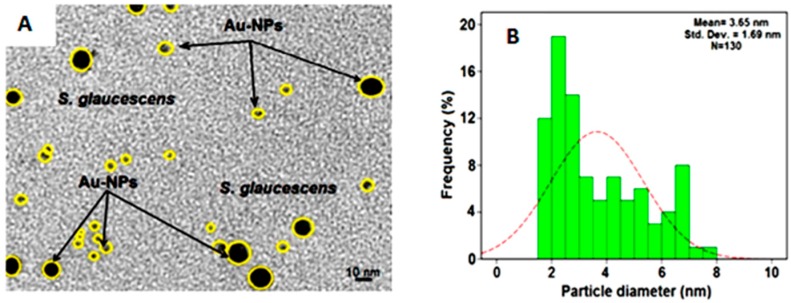
TEM image (**A**); Corresponding size distribution (**B**) of Au/SG after 4 h of stirring times.

To determine the ultrastructural morphology of AuNPs, a drop of the solution of AuNPs synthesized by treating gold(III) chloride trihydrate solution with SG was deposited onto a transmission electron microscope (TEM) copper grid, dried, and the grid imaged (Figure 4A). The results showed that AuNPs had particle size distributions with a mean diameter of 3.65 ± 1.69 nm (Figure 4B). Figure 4A also shows the AuNPs surrounded by the SG extract. The dark points in this figure represent the large scale distribution of AuNPs. The AuNPs was approximately 130 in number.

**Figure 5:**
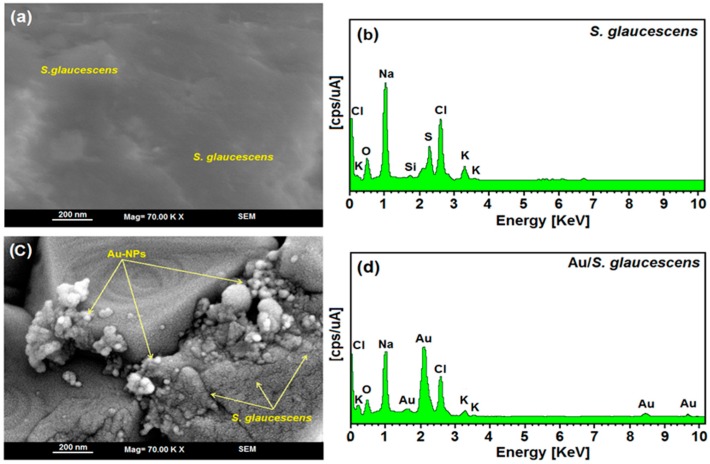
SEM image and EDXRF spectra of SG and Au/SG formation after 4 h of reaction time (**a**–**d**).

Figure 5a–d show the SEM images and EDXRF spectrum for the SG and Au/SG suspension after 4 h of stirring. The structure of SG extract without AuNPs showed a large mass with a specific form, that is the typical shape of the plant extract. The surface structure of Au/SG with or without AuNPs showed a large mass that is typical of the plant extract structure.

The exterior surfaces of Au/SG appeared shiny because of the presence of small AuNPs (Figure 5c). Figure 5b–d shows the EDXRF spectra for the SG and SG-stabilized AuNPs; the peaks at approximately 0.11, 0.23, 0.56, 1.05, 1.78, 2.35, 2.62, 3.37, and 3.59 KV correspond to the binding energies of SG and in Figure 5d, the peaks at approximately 1.65, 2.15, 8.44, and 9.73 KV correspond to the gold elements in the SG.

### 2.3. Anticancer Activity of SG-Stabilized AuNPs

#### 2.3.1. Cytotoxicity ASSAY

**Figure 6:**
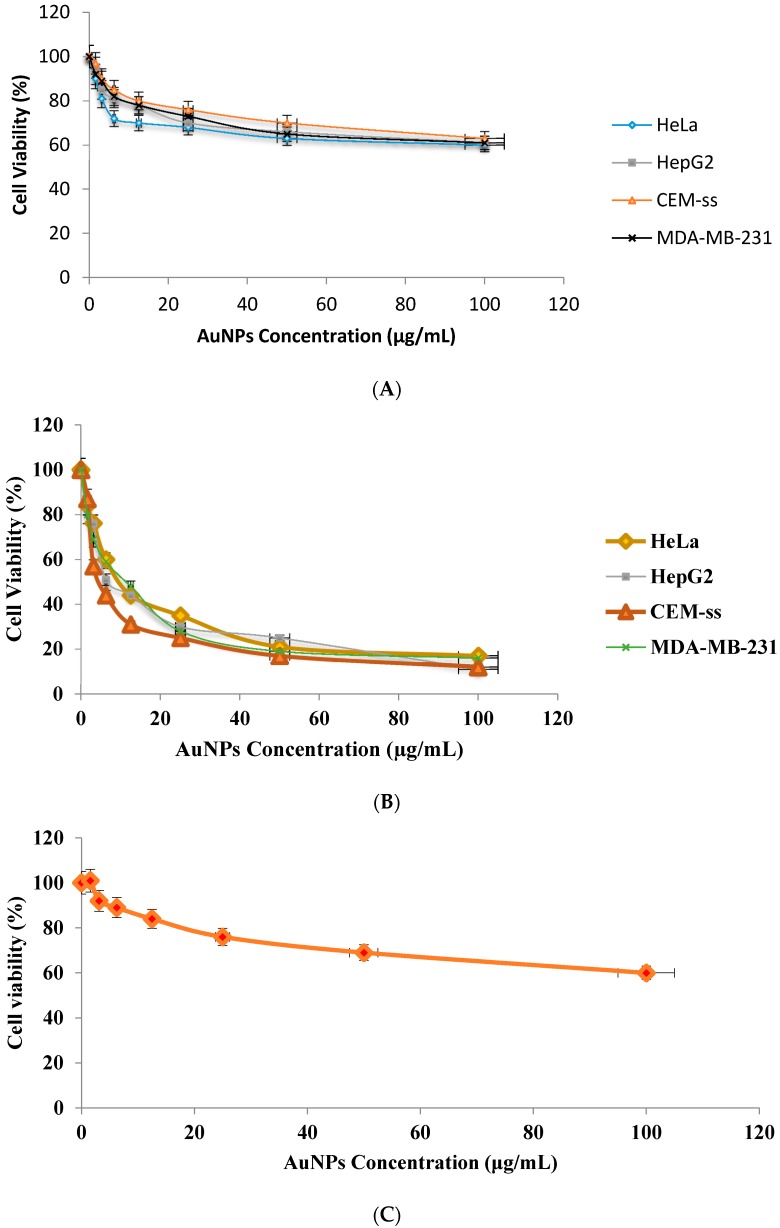
(**A**) Cytotoxic effect of SG alone on various cancer cells at 72 h of treatment; (**B**) Cytotoxic effect of SG-stabilized AuNPs on various cancer cells at 72 h of treatment were evaluated through mitochondrial activity using the MTT assay; (**C**) Cytotoxic effect of SG-stabilized AuNPs on normal human mammary epithelial cells (MCF-10A) at 72 h of treatment were evaluated by MTT assay (**B**). Each point is the mean value of three replicates.

The cytotoxicity assay showed that SG alone was not toxic (*p* > 0.05) to the various cancer cell lines in a dose- and time-dependent manner (Figure 6A), whereas SG-stabilized AuNPs significantly (*p <* 0.05) inhibited the proliferation of the various cancer cell lines in a dose- and time-dependent manner (Figure 6B). However, the SG-stabilized AuNPS were not toxic (*p* > 0.05) to the normal human mammary epithelial cells (MCF-10A) (Figure 6C). The IC_50_ values for SG-stabilized AuNPs on cancer cells and were 4.75 ± 1.23 μg/mL (HeLa), 7.14 ± 1.45 μg/mL (HepG2), 10.32 ± 1.5 μg/mL (CEM-ss) and 11.82 ± 0.9 μg/mL (MDA-MB-231) after 72 h of treatment. Based on the IC_50_, HeLa cells were selected for further investigation.

#### 2.3.2. Apoptosis Detection Assay

**Figure 7:**
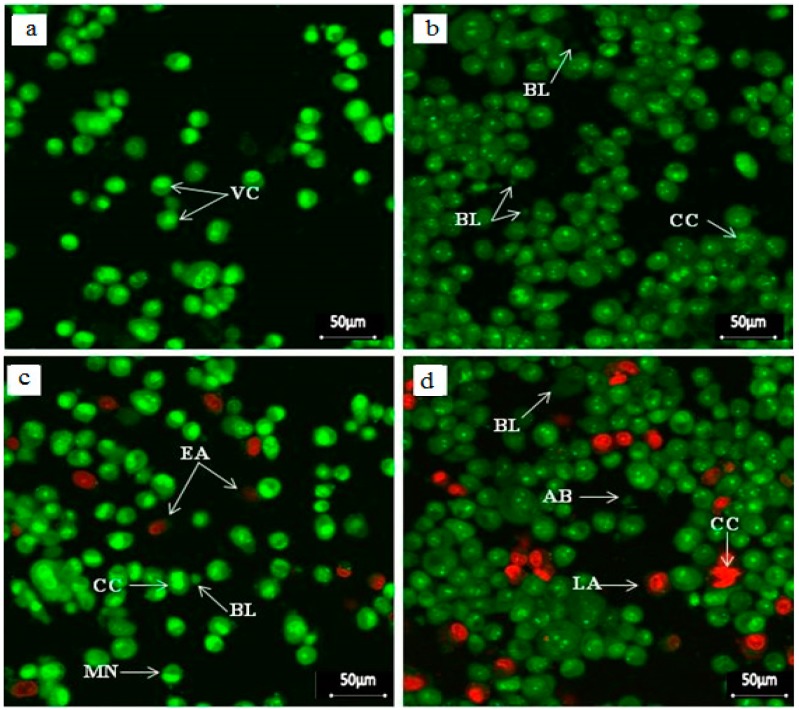
Fluorescent micrographs of AO/PI double stained HeLa cells that were treated with were 4.75 ± 1.23 μg/mL SG-stabilized AuNPs; (**a**) Untreated cells showing normal cell structures; (**b**) Treated cells after 24 h incubation showing membrane blebbing and chromatin condensation; (**c**) Membrane blebbing, chromatin condensation and nuclear margination with early apoptotic cells after 48 h treatment; (**d**) Membrane blebbing, chromatin condensation and apoptotic body formation with numerous late apoptotic cells after 72 h treatment. VC: Viable cells, EA: Early apoptotic cells, CC: Chromatin condensation, BL: Membrane blebbing, MN: Marginated nucleus, LA: Late apoptotic cells, and AB: Apoptotic body (400× magnification).

Apoptosis induction is the desired mode of cell death in the treatment of cancers. In the current study, distinct morphological changes, such as cell shrinkage, chromatin condensation, membrane blebbing, nuclear margination and apoptotic body formation were exhibited in SG-stabilized AuNPs treated HeLa cells after several periods of treatment (Figure 7a–d).

#### 2.3.3. Annexin V-FITC Assay

**Figure 8:**
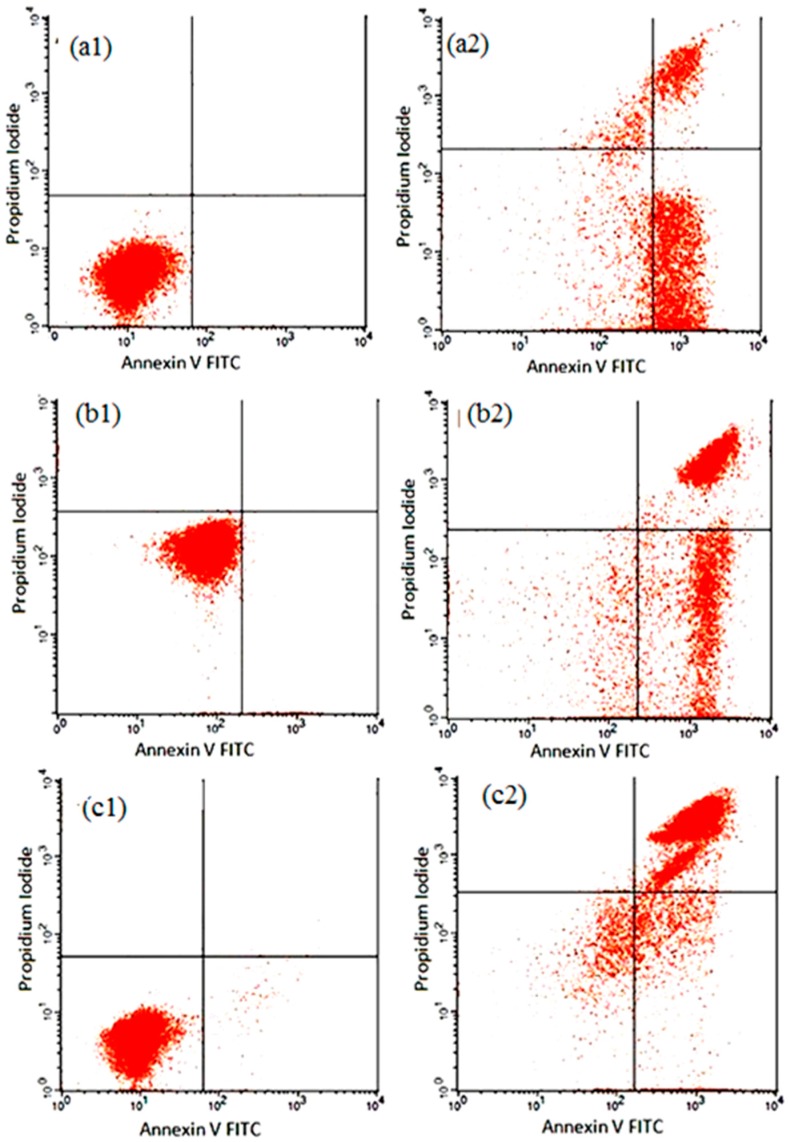
Flow cytometric analysis of apoptosis induction by 4.75 ± 1.23 μg/mL SG-stabilized AuNPs in HeLa cells after staining with FITC-conjugated annexin-V and PI. (**a1**–**c1**) Untreated (control) HeLa cells at 12, 24 and 48 h incubation respectively; (**a2**–**c2**) Effects of 12, 24 and 48 h SG-stabilized AuNPs treatment, respectively.

The early apoptotic effect of SG-stabilized AuNPs was further ascertained by determining the percentage of apoptotic cells using annexin V-FITC staining and flow cytometric analysis. In this study, SG-stabilized AuNPs clearly reduced the percentage of viable cells, while significantly (*p <* 0.05) increased the percentage of early and late apoptotic cells at 12, 24, and 48 h of treatment, respectively (Figure 8 and Table 1. Thus, the results further showed that 4.75 ± 1.23 μg/mL SG-stabilized AuNPs have antiproliferative effects towards HeLa cells through the induction of apoptosis.

#### 2.3.4. Cell Cycle Assay

**Figure 9:**
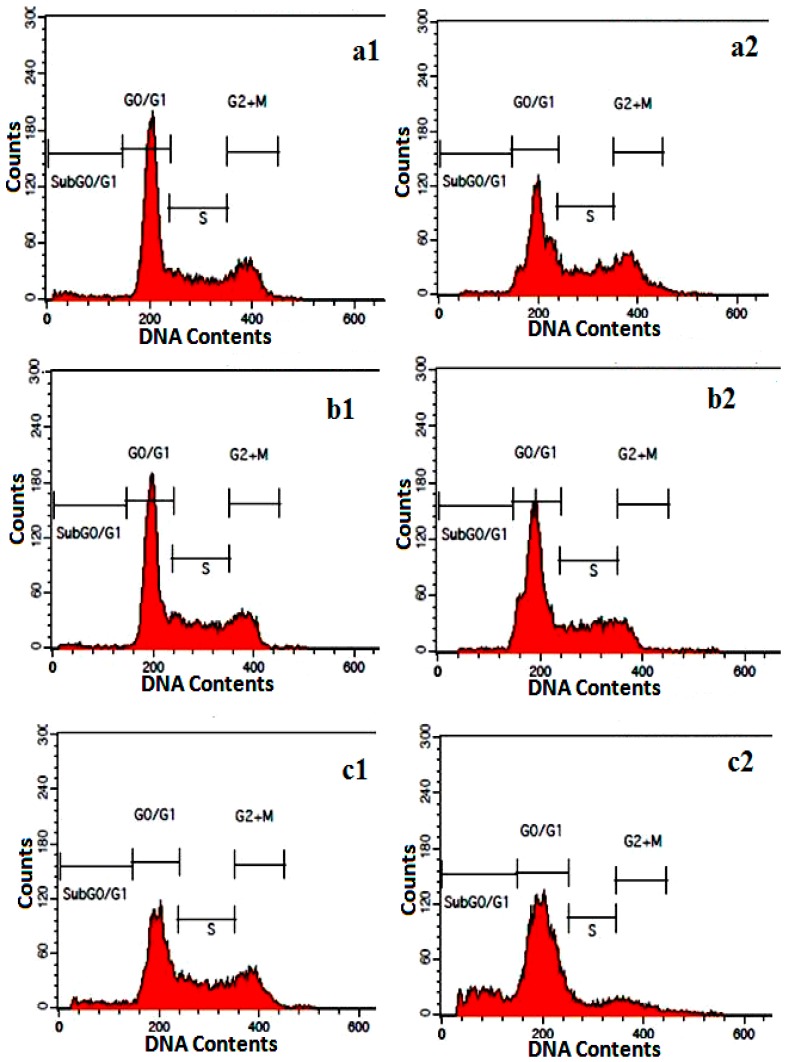
Cell cycle analysis of HeLa cells treated with 4.75 ± 1.23 μg/mL SG-stabilized AuNPs after staining with PI. (**a1**–**c1**) Untreated HeLa cells for 24, 48 and 72 h respectively; (**a2**–**c2**) Effects of 24, 48 and 72 h, respectively exposure of HeLa cells to SG-stabilized AuNPs. G0/G1, G2/M, and S indicate the cell phase, and sub G0–G1 refers to the portion of apoptotic cells.

In the cell cycle analysis, using apoptosis-inducing agents, a sub-G1 cell population, which indicated apoptotic cells appeared that is believed to be the result of endonuclease activation and subsequent leakage of DNA from the cells. As shown in Figure 9 and Table 2, the sub-G1 population increased in a time-dependent manner from 11.10 ± 0.98, to 19.0 ± 0.40, and 24.85% ± 1.56% after exposure to SG-stabilized AuNPs for 24, 48 and 72 h, respectively. There was also an accumulation of DNA fragmented cells in the G2/M phase (Figure 9 and Table 2).

**Table 1:** Flow cytometric analysis of HeLa cells after treating with Au/SG–NPs. The cells were stained with FITC-conjugated annexin-V and PI and incubated at 37 °C for 12, 24 and 48 h. Values are expressed as mean ± SD of three different experiments. The data has been analyzed using *post hoc* comparison test-one way ANOVA, means compared with Tukey’s-b test. (*): significant (*p* < 0.05) increasing of early apoptotic cells in SG-stabilized AuNPs treated groups in comparison to that of untreated controls. (**): significant (*p* < 0.05) increasing of late apoptotic/necrotic cells in SG-stabilized AuNPs treated groups in comparison to that of untreated controls.

Cells (%)						
Cell Condition	Control 12 h	Treated 12 h	Control 24 h	Treated 24 h	Control 48 h	Treated 48 h
**Viable Cells**	95.04 ± 0.71	84.59 ± 0.56	90.33 ± 0.50	68.29 ± 0.86	87.18 ± 0.26	54.08 ± 1.33
**Early Apoptosis**	1.5 ± 0.14	5.75 ± 0.61 *	1.88 ± 0.57	9.50 ± 1.38 *	3.50 ± 2.41	14.04 ± 1.52 *
**Late Apoptosis/Necrosis**	3.5 ± 1.24	7.73 ± 0.65 **	7.79 ± 0.20	22.21 ± 0.72 **	9.32 ± 1.25	31.88 ± 0.40 **

**Table 2:** Flow cytometric analysis of HeLa cells after treating with 4.75 ± 1.23 μg/mL SG-stabilized AuNPs. The cells were stained with PI and incubated at 37 °C for 24, 48 and 72 h. Values are expressed as mean ± SD of three different experiments. The data has been analyzed using *post hoc* comparison test-one way ANOVA, means compared with Tukey’s-b test. (*): significant (*p* < 0.05) increasing of cells in Sub G0/G1 phase in SG-stabilized AuNPs treated groups in comparing to that of untreated controls.

Cell Cycle Phases			Cells (%)			
	Control 24 h	Treated 24 h	Control 48 h	Treated 48 h	Control 72 h	Treated 72 h
**G0/G1**	55.25 ± 0.6	32.44 ± 0.95	51.15 ± 0.25	43.61 ± 0.55	36.64 ± 0.35	46.68 ± 1.68
**G2/M**	18.85 ± 0.46	19.29 ± 0.11	20.16 ± 0.29	11.29 ± 0.39	25.22 ± 0.25	9.06 ± 1.93
**Synthesis**	23.91 ± 0.25	37.23 ± 0.63	28.24 ± 0.86	23.93 ± 0.32	36.95 ± 0.65	20.35 ± 1.18
**Sub G0/G1**	1.98 ± 0.75	11.10 ± 0.98 *	1.20 ± 0.24	19.0 ± 0.40 *	2.02 ± 1.46	24.85 ± 1.56 *

#### 2.3.5. Caspase Assay

**Figure 10:**
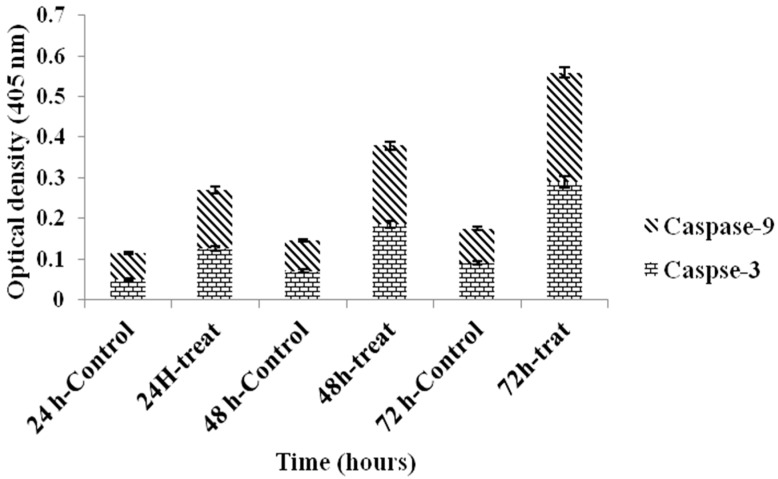
Effect of 4.75 ± 1.23 μg/mL SG-stabilized AuNPs treatment on HeLa cell caspase-3 and -9. The values are mean % ± SD of three independent experiments. Significant differences (*p <* 0.05) between treated and control groups for caspase-3 and -9 is found.

The effect of 4.75 ± 1.23 μg/mL SG-stabilized AuNPs on apoptosis of HeLa cells were also determined by estimating caspase-3 and -9 activities. The caspase-3 and -9 activities increased significantly (*p <* 0.05) in a time-dependent manner (Figure 10 and Table 3).

**Table 3:** Caspases spectrophotometric analysis of HeLa cells after treating with SG-stabilized AuNPs for 24, 48 and 72 h. Values are expressed as mean ± SD of three different experiments. The data has been analyzed using *post hoc* comparison test-one way ANOVA, means compare with Tukey’s-b test. (*): significant (*p* < 0.05) increasing of apoptotic cells in SG-stabilized AuNPs treated groups in comparing to that of untreated controls.

Cells %						
Caspase	Control 24 h	Treated 24 h	Control 48 h	Treated 48 h	Control 72 h	Treated 72 h
**Caspase-3**	0.05 ± 0.3	0.125 ± 2.2 *	0.07 ± 0.15	0.185 ± 0.5 *	0.09 ± 1.91	0.29 ± 0.55 *
**Caspase-9**	0.065 ± 0.27	0.145 ± 0.9 *	0.075 ± 0.75	0.195 ± 0.65 *	0.085 ± 1.33	0.27 ± 1.15 *

## 3. Discussion

Nanotechnology is gaining recognition as an important component of modern biomedical research, particularly in cancer detection, diagnosis, and therapy. However, in comparison with other technologies, nanotechnological research is still relatively in its infancy [7].

In cancer treatments, effective drug-delivery has been a concern because the biological and physiological barriers provided by cancer tissues that challenge the efficacy of even the most potent drugs. There is a great need for the development of new and innovative drug to overcome the barriers imposed by cancers [7]. It is proposed that nanoparticle systems could be effective in the treatment of cancers. In the current study, we propose that the SG-stabilized AuNPs have anticancer properties. The SG-stabilized AuNPs were synthesized from seaweeds using a fast, single step and completely green biosynthetic method.

Gold nanoparticles can be manufactured into a variety of shapes including gold nanospheres, nanorods, nanobelts, nanocages, nanoprisms, and nanostars [23]. The size and shape of gold nanoparticles strongly influence their chemical, optical, and electromagnetic properties. For instance, metallic gold is golden yellow, gold nanorods are blue or black in solution while spherical gold nanoparticles have a visible red wine color [6,12]. In this study, after addition of HAuCl4 into SG water extract and stirring, a reddish suspension resulted, which suggests the synthesis of gold nanoparticles in the suspension. The SG extract yielded 47.83% of AuNPs with high-purity and the SG-stabilized AuNPs were in a narrow size distribution range. The shape and size the SG-stabilized AuNPs were stable. These properties suggest that if their biological properties can be established, the SG-stabilized AuNPs have great potential to be developed into a therapeutic compound.

Over the past two decades, research on nanoscale matter has made rapid progress and now the structure, composition, morphology, size, and shape of the nanoscale matter can sufficiently be controlled by manipulating the production processes [6]. In our study, we believe that SG-stabilized AuNPs from seaweeds also have those properties and in addition the nanoparticles are non-toxic to normal cells. The method of AuNPs production is relative simple and repeatable, thus easy to scale-up and inexpensive to produce.

Various biomedical applications have been suggested for AuNPs [24,25,26]. We propose that SG-stabilized AuNPs have effective anticancer effects. To demosntrate the anticancer properties of the nanoparticles, an *in vitro* study was conducted to determine their effect on cancer cells. The results showed that SG-stabilized AuNPs at various concentrations significantly inhibited cancer cell lines, namely breast, liver, cervical, and leukemia cell lines. These findings suggest that the effect of AuNPs is not specific for a particular cancer, but rather they can potentially inhibit growth of several cancers without adversely affecting normal cells [27,28,29].

It has been suggested that nanoparticles may cause some cytotoxicity in cell culture medium through their adherence to the cell membrane, internalization, and degradation [30]. In our study, SG-stabilized AuNPs were most cytotoxic to HeLa cells, the cell line chosen for further experimentation. Although very small sized gold nanoparticles are not cytotoxic *in vitro* [31], intracellular AuNPs could cause changes in morphology, spreading, migration, and protein production of HeLa cells.

The antiproliferative effect of SG-stabilized AuNPs on HeLa cells was determined by induction of apoptosis and change of cellular morphology. The study showed that AuNPs induce apoptosis of HeLa cells after 24, 48 and 72 h incubation. Membrane blebbing and chromatin condensation after 24 h, membrane blebbing, chromatin condensation and nuclear margination with early apoptotic cells after 48 h, and membrane blebbing, chromatin condensation, and apoptotic body formation with numerous late apoptotic cells after 72 h treatments, were observed. In contrast, the treated normal cells showed normal morphology. Thus, the results suggest that the apoptotic effect of AuNPs was time-dependent and cancer cell specific.

The morphological changes in HeLa cells induced by AuNPs was supported by cell cycle analysis using flow cytometry after AO/PI and Annexin-V/PI staining that allowed for the detection, quantification, and discrimination between apoptotic, necrotic, and dead cells [32]. Cell cycle evaluation, shifts in distribution cell cycle phases in response to stimuli, growth factors, drugs, mutations or nutrients can readily be assessed by flow cytometry via the stain of DNA using dyes such as PI [33]. In the present study, the percentage of viable HeLa cells gradually decreased with increase in early and late apoptotic cell population after SG-stabilized AuNPs treatment. The cell cycle analysis demonstrated that SG-stabilized AuNPs was induced significant (*p* < 0.05) accumulation of apoptotic HeLa cells in the sub-G0/G1 phase and cell cycle arrest at the G2/M phase after 24, 48 and 72 h of treatment. These results confirmed that the death of SG-stabilized AuNPs-treated HeLa cells was via apoptosis [34].

The activation of caspase proteases is a critical event in the induction of apoptosis. Caspase is produced as inactive zymogens and undergo proteolytic activation during apoptosis. Caspase-9 is upstream initiator caspase, while caspase-3 is one of the downstream effector that plays a central role in the initiation of apoptosis [35]. The outcomes of this study showed time-dependent increase in caspase-3 and -9 activities in HeLa cells after treatment with SG-stabilized AuNPs. Thus, the apoptotic cell death of HeLa as the result of SG-stabilized AuNPs treatment is via the intrinsic pathway.

Although gold may have lost its early promise as a therapeutic agent, its use in nanoparticles is set to revive its applications in medicine, both in the treatment and diagnosis of diseases. In the form of AuNPs, gold can be applied intramuscularly, intravenously, or orally to treat diseases like cancers [12]. Although, applications of metal nanoparticles in biomedicine are relatively unknown, recent discoveries may eventually realise the potential of these nanoparticles as therapeutic compounds in the treatments of diseases such as cancers.

## 4. Materials and Methods

### 4.1. Chemicals

The brown seaweed SG was collected from coastal areas of Chabahar (Sistan and Balochestan, Iran). Hydrogen tetrachloroaurate (III) (HAuCl_4_. 3H_2_O, 99.98%) used as a gold precursor, HNO_3_ (70%) and HCl (37%) mwer obtained from Sigma-Aldrich (St. Louis, MO, USA). All reagents in this study were analytical grade and were used as received without further purification. All solutions were freshly prepared using double distilled water and kept in the dark to avoid any photochemical reactions. Glassware used in experimental procedures were cleaned in a fresh solution of HNO_3_/HCl (3:1, *v*/*v*), washed thoroughly with double-distilled water, and dried before use.

### 4.2. Cell Culture

Human leukemia cell line (CEM-ss), breast cancer cell line (MDA-MB-231), cervical cancer cell line (HeLa), liver cancer cell line (HepG2), and normal human mammary epithelial cell line (MCF-10A) were purchased from American Type Culture Collection (ATCC) (Maryland, MD, USA). The cells were maintained in RPMI-1640 (ATCC) medium supplemented with 10% heat inactivated fetal bovine serum (FBS) (ATCC), and a 1% mixture of penicillin/streptomycin and amphotericin B (Sigma-Aldrich). All other used chemical and reagents were high grade.

### 4.3. SG Extract Preparation

The fresh, dry powder and SG water extract are shown in Figure 1a–c. The SG was washed with water to remove adhering mud particles and impurities and freeze-dried for two days to completely remove the moisture, and stored at −20 °C. The seaweed was cut into small pieces, powdered and then sieved using a 20 mesh sieve to obtain particles of uniform size that was used in the study. To produce the water extract, SG powder (4.0 g) was added to a 200 mL Erlenmeyer flask containing sterile distilled water (100 mL) and then mixed for 4 h at 80 °C temperatures.

### 4.4. Synthesis of Au/SG Suspension

Briefly, SG water extract (10 mL) was added to distilled, deionized water (50 mL) with vigorous stirring. Hydrogen tetrachloroaurate(III) (HAuCl_4_·3H_2_O, 20 mL, 5 × 10^−3^ M) was then added and mixed for 4 h at room temperature to obtain the AuNPs. Throughout the reduction process, the solution was kept at a room temperature in the dark to avoid a photochemical reaction. The suspension was purged with nitrogen gas to eliminate oxygen. The resultant colloidal Au/SG suspensions were then centrifuged at 15,000× *g* for 20 min and washed four times to remove gold ion residues. The precipitated nanoparticles were dried overnight at 30 °C under vacuum to obtain the SG-stabilized AuNPs powder.

### 4.5. Characterization of SG-Stabilized AuNPs

The SG-stabilized AuNPs suspension was characterized by ultraviolet-visible spectroscopy (UV-Vis), transmission electron microscopy (TEM), scanning electron microscopy (SEM), and energy dispersive X-ray fluorescence spectrometry (EDXRF). The UV-visible spectra were recorded over the 300–1000 nm range with a UV 1650 PC-Shimadzu B UV-visible spectrophotometer (Shimadzu, Osaka, Japan). TEM observations were carried out on a H-7100 electron microscope (Hitachi, Tokyo, Japan), and the particle size distributions determined using the UTHSCSA Image Tool version 3.0 program. SEM was performed using a Philips XL-30 instrument (Philips, Eindhoven, The Netherlands) to examine the morphology of Au/SG. The EDXRF was carried out on a DX-700HS spectrometer (Shimadzu).

### 4.6. Determination of Anticancer Activity

#### 4.6.1. Cytotoxicity Assay

The antiproliferative effect of SG-stabilized AuNPs was determined by MTT (3-[4,5-dimethylthiazol-2-yl]-2,5 diphenyl tetrazolium bromide) assay [36]. The cells were allowed to grow until 90% confluency and cell density determined using a hemocytometer (Marienfeld, Lauda-Königshofen, Germany). Then, cell suspension was seeded into each well of 96-well microculture plates (TPP, Trasadingen, Switzerland) at a concentration of 2 × 10^5^ cells/well and treated with various concentrations of SG-stabilized AuNPs. After incubation for 72 h at 37 °C, MTT solution (Sigma-Aldrich) in phosphate buffered saline (PBS, pH 7.5) (Sigma-Aldrich) was added to each well, and the plate incubated for an additional 4 h in the dark. The media were then aspirated and replaced with same volume of dimethyl sulfoxide (DMSO) (Sigma-Aldrich). Experiments were performed in triplicates. Consequently, the optical density (OD) was measured at 570 nm using an enzyme-linked immunosorbent assay (ELISA) plate reader (Universal Microplate reader, Biotech, Inc, 100 Tigan Street, Winooski, VT, USA). The IC_50_ value was determined from absorbance *vs.* concentration curve. Finally, the values were compared to those of the positive antineoplastic agent, doxorubicin (Sigma-Aldrich) with DMSO (Sigma Aldrich) (0.1%) as a negative control.

#### 4.6.2. Apoptosis Detection Assay

Morphological changes of HeLa death cells induced by SG-stabilized AuNPs were evaluated using the AO/PI (acridine orange/propidium iodide) double staining method according to the standard procedure and were examined under a fluorescence microscope (Zeiss, Deutschland, Germany). The HeLa cells were plated at a concentration of 1 *×* 10^6^ cells/mL, treated with SG-stabilized AuNPs at several concentrations and incubated under CO_2_ at 37 °C, for 24, 48, and 72 h. After treatment, the cells were centrifuged at 200× *g* for 10 min and the supernatant discarded. Then, the cells were washed twice with phosphate buffer solution (PBS, pH 7.4) after centrifuge at 200× *g* for 10 min to remove the remaining media. 10 µL of the cell pellets were stained with 10 μL of a fluorescent dye mixture containing equal volumes (100 µg/mL) of AO and PI for 2 min. Approximately 10 μL of freshly stained cell suspension was placed onto a glass slide, covered with cover slip and examined under a fluorescence microscope within 30 min before the fluorescence begins to fade [37]. Experiments were performed in triplicates.

#### 4.6.3. Annexin V-FITC Assay

Apoptosis of HeLa cells treated with SG-stabilized AuNPs was determined with an annexin V-FITC kit (Sigma Aldrich). The HeLa cells (1 × 10^6^ cells/mL) were treated with SG-stabilized AuNPs for 6, 12 and 24 h. Untreated cells were used as control. The HeLa cells were then collected and centrifuged at 200× *g* for 10 min to remove the media. The cell pellets were washed twice with 1 mL of ice-cold PBS (7.4) and re-centrifuged at 200× *g* for 10 min and re-suspended in ice-cold 1× binding buffer at a density of 1 × 10^6^ cells/mL. Precisely 500 μL of cell suspension was transferred to a test tube, to which 5 μL of annexin V-FITC conjugate and 10 μL of PI were added. The cells were gently vortexed and incubated for 15 min at room temperature in the dark. Finally, flowcytometric analysis was conducted under laser emitting excitation light at 488 nm using a BD FACS Calibur flow cytometer equipped with an argon laser (BD, 2350 Qume Drive, San Jose, CA, USA). Data analysis was performed using the BD CellQuest Pro software (version 5.1).

#### 4.6.4. Cell Cycle Assay

Cell cycle analysis by means of flow cytometry was used to further ascertain the cytotoxicity of SG-stabilized AuNPs towards HeLa cells. Approximately 2.5 × 10^6^ cells/mL HeLa cells were cultured with SG-stabilized AuNPs and incubated in a CO_2_ incubator at 37 °C, for 24, 48 and 72 h. The cells were harvested by centrifugation at 1500× *g* at 4 °C for 5 min and washed with 1 mL washing PBS (pH, 7.4) containing 0.1% sodium azide. Subsequently, 80% ice-cold ethanol (500 µL) was were added drop by drop to the cell pellets with continuous vortex-mixing to prevent clumping and aggregation of cells, and then kept at −20 °C for 48 h. Then, PBS (1 mL, pH 7.4) was then added and centrifuged at 1500× *g* and 4 °C for 5 min to remove the ethanol. The cell pellets were washed twice with washing PBS (1 mL) and stained with PBS staining buffer containing 50 µg/mL RNAase A and 3 µg/mL PI and incubated in the dark on the ice for 20 min. Flow cytometric analysis was conducted by laser emitting excitation light at 488 nm using BD FACS Calibur flow cytometer equipped with an argon laser. Data analysis was performed using the BD CellQuest Pro software (version 5.1).

#### 4.6.5. Caspase Assays

The activities of caspases -3 and -9 in HeLa cells were determined using a colorimetric assay kit (Gene Script Kit, Corporation Inc., Piscataway, NJ, USA). A cell suspension containing about 2 × 10^6^ HeLa cells/mL was treated with SG-stabilized AuNPs and the untreated cells as control were incubated under CO_2_ at 37 °C for 24, 48 and 72 h. The cells were centrifuged for 5 min at 2000× *g* and 4 °C to remove the media and then washed twice with PBS and re-centrifuged at 2000× *g* and 4 °C for 5 min. The cell pellets were lysed with cold-prepared lysis buffer (50 µL) containing dithiothreitol (DTT, 0.5 µL) and phenylmethylsulfonyl fluoride (PMSF, 0.25 µL), mixed well and incubated on ice for exactly 1 h with continuous vortexing 3 to 4 times for 10 s. The resulting cell lysate was centrifuged for 1 min at 10,000× *g* and 4 °C to obtain the supernatant. Protein concentration in each supernatant was quantified using the Bradford method [38]. Fifty µL of 2× reaction buffer containing DTT (0.5 µL) and PMSF (0.25 µL) were then added to supernatant (50 µL) containing protein (200 µg). Then, caspase substrates (5 µL) were added and the mixture transferred to a 96-well plate (TPP), incubated at 37 °C in the dark for 4 h. At the end of the incubation period, the samples were read at 405 nm in a microplate reader (Biotech, Inc., Oklahoma City, OK, USA). Data was presented as optical density (OD) (405 nm; mean ± SD) and histogram plotted.

### 4.7. Statistical Analysis

The experiments were conducted in triplicate and the results expressed as mean ± S.D. Statistical analysis was done using SPSS version 20.0 (SPSS Inc., 233 South Wacker Drive, 11th Floor, Chicago, IL, USA). Data have been analyzed using *post hoc* comparison test-one way analysis of variance (ANOVA), means compared with Tukey’s-b test. Probability values of less than alpha 0.05 (*p <* 0.05) were considered statistically significant.

## 5. Conclusions

Most chemotherapeutic used in the treatment of cancers are plagued with toxic side-effects that sometimes even cause fatalities. The SG-stabilized AuNPs produced from seaweed SG do not show these side-effects and does not affect normal cells adversely. Production of SG-stabilized AuNPs can be achieved by a reliable eco-friendly process, while being an important contribution to the development of nanotechnology, is also a technique in gold nanoparticle synthesis with vast potentials for pharmaceutical applications. The process of production of gold nanostructures from seaweed SG through an efficient green chemistry process avoids the use of hazardous and toxic solvents and the production of waste. This gold nanoparticles production technique is also relative simple, inexpensive and easily up-scalable, which are properties that can be beneficial in the production of therapeutic compounds for human use.
